# Supplementary material for: Functional brain networks reveal the existence of cognitive reserve and the interplay between network topology and dynamics
Source: Sci Rep. 2018 Jul 12;8:10525. doi: 10.1038/s41598-018-28747-6 (PMC6043549; doi:10.1038/s41598-018-28747-6)
Supplement: Supplementary file 1 — Supplementary information [file 41598_2018_28747_MOESM1_ESM.docx]

**TITLE PAGE**

TITLE: **Functional brain networks reveal the existence of cognitive reserve and the interplay between network topology and dynamics**

RUNNING TITLE: Topological and dynamical networks in cognitive reserve

AUTHORS: Johann H. Martínez^1,2^ *, María Eugenia López^3^ *, Pedro Ariza^2^, Mario Chavez^4^, José A. Pineda-Pardo^5,6^, David López-Sanz^3,7^, Pedro Gil^8,9^, Fernando Maestú^3,7^ and Javier M. Buldú^2,10^.

### * These authors have contributed equally and must be considered as the first authors

### ^1^ INSERM, Institut du Cerveau et de la Moelle Epinière (ICM). Hôpital Pitié Salpétrière, Paris, France

^2^ Laboratory of Biological Networks, Center for Biomedical Technology (CTB), Madrid, Spain

^3^ Department of Basic Psychology II, Complutense University of Madrid, Madrid, Spain

^4^ 7CNRS-UMR 7225, Hôpital Pitié-Salpetrière, Paris, France.

^5^ Centro Integral de Neurociencias AC (CINAC), HM Puerta del Sur, Madrid, Spain

^6^ CEU San Pablo University, Madrid, Spain.

^7^Laboratory of Cognitive and Computational Neuroscience (UCM-UPM), Center for Biomedical Technology (CTB), Madrid, Spain

^8^ Institute of Sanitary Investigation [IdISSC], San Carlos University Hospital, Madrid, Spain

^9^ Geriatrics Department, San Carlos University Hospital, Madrid, Spain.

^10^ Complex Systems Group, Universidad Rey Juan Carlos, Madrid, Spain

1. Johann H. Martínez*

### INSERM, Institut du Cerveau et de la Moelle Epinière (ICM). INSERM U11127 ICM-Hôpital Pitié Salpétrière. 47 Bd de l'Hopital PC: 75013, Paris, France,

and Laboratory of Biological Networks, Center for Biomedical Technology, UPM, Campus de Montegancedo, 28223, Pozuelo de Alarcón, Madrid, Spain

### Email: [johemart@gmail.com](mailto:johemart@gmail.com)

Telephone Number: (+33) 0625708097

Contribution(s): Contributed writing paper, in-silico experimental setup, wrote code for results, analysed the data

1. María Eugenia López* (corresponding autor)

Department of Basic Psychology II, Complutense University of Madrid, Campus de Somosaguas 28223, Madrid, Spain

Email: [meugenia.lopez@ctb.upm.es](mailto:meugenia.lopez@ctb.upm.es)

Contribution(s): Magnetoencephalographic recordings and writing paper

1. Pedro Ariza

Laboratory of Biological Networks, Center for Biomedical Technology, UPM, Campus de Montegancedo, 28223, Pozuelo de Alarcón, Madrid, Spain

Email: [pedro.ariza@ctb.upm.es](mailto:pedro.ariza@ctb.upm.es)

Telephone number: [+34 6](tel:+34%20659%2040%2018%2051)17039770

Contribution(s): Contributed with the analysis of the data

1. Mario Chavez

Hôpital Pitié-Salpetrière, 7CNRS-UMR 7225. Paris, France.

Contribution(s): MC contributed with results and statistical analysis

Email: [mario.chavez@upmc.fr](file:///D:\%22mailto)

1. José A. Pineda- Pardo

Centro Integral de Neurociencias AC (CINAC), HM Puerta del Sur, Hospitales de Madrid Mostoles, 28938 Madrid, Spain

CEU San Pablo University, Campus de Moncloa, Calle Julián Romea, 18, 28003 Madrid, Spain.

Email: [joseangel.ppardo@gmail.com](mailto:joseangel.ppardo@gmail.com)

Telephone Number [+34 659401851](tel:+34%20659%2040%2018%2051)

Contribution(s) Statistical analysis

1. David López-Sanz

Laboratory of Cognitive and Computational Neuroscience (UCM-UPM), Centre for Biomedical Technology (CTB), Campus de Montegancedo, 28223 Pozuelo de Alarcón, Madrid, Spain;

and Department of Basic Psychology II, Complutense University of Madrid, Campus de Somosaguas 28223, Madrid, Spain.

Email: [david.lopez@ctb.upm.es](mailto:david.lopez@ctb.upm.es)

Telephone Number: [(+34) 91 3364642](tel:913%2036%2046%2042)

Contribution(s): Contributed writing the paper

1. Pedro Gil

Institute of Sanitary Investigation [IdISSC], San Carlos University Hospital, Calle del Prof Martín Lagos, s/n, 28040 Madrid, Spain;

and Geriatrics Department, San Carlos University Hospital, Calle del Prof Martín Lagos, s/n, 28040, Madrid, Spain.

Email: [pgil@salud.madrid.org](mailto:pgil@salud.madrid.org)

Contribution(s): Contributed with the clinical assessment of the participants

1. Fernando Maestú

Laboratory of Cognitive and Computational Neuroscience (UCM-UPM), Centre for Biomedical Technology (CTB), Campus de Montegancedo, 28223 Pozuelo de Alarcón Madrid, Spain;

and Department of Basic Psychology II, Complutense University of Madrid, Campus de Somosaguas 28223, Madrid, Spain.

Email: [fernando.maestu@ctb.upm.es](mailto:fernando.maestu@ctb.upm.es)

Contribution(s): Design the paradigm

1. Javier M. Buldú

Complex Systems Group, Universidad Rey Juan Carlos, Calle Tulipán s/n, 28933, Móstoles, Spain, Madrid, Spain;

and Laboratory of Biological Networks, Center for Biomedical Technology, UPM, Campus de Montegancedo, 28223 Pozuelo de Alarcón, Madrid, Spain.

Email: [javier.buldu@urjc.es](mailto:javier.buldu@urjc.es)

Telephone number: [+34 6](tel:+34%20659%2040%2018%2051)17039770

Contribution(s): Conceived the analysis, analyzed the data and wrote the manuscript.

**SUPLEMENTARY INFORMATION**

**S1.- DEFINITION OF NETWORK METRICS**

The strength ***s(i)*** of a node *i* is the sum of the weights $w_{ij}$ of all its links:

$$\boldsymbol{s}\left( \boldsymbol{i} \right)\boldsymbol{=}\sum_{\boldsymbol{j\in N}} \boldsymbol{w}_{\boldsymbol{ij}}$$

The strength ***S*** of a functional network is the average of the strength of all its nodes.

The strength of the nearest neighbours ***snn(i)*** as the average strength of all neighbours of node *i*.

The outreach ***o(i)*** of a node *i* is the sum of the links’ weights $w_{ij}$ multiplied by the link

Euclidean lengths $l_{ij}$ between node *i* and node *j*:

$$\boldsymbol{o}\left( \boldsymbol{i} \right)\boldsymbol{=}\sum_{\boldsymbol{j\in N}} \boldsymbol{l}_{\boldsymbol{ij}}\boldsymbol{w}_{\boldsymbol{ij}}$$

The outreach ***O*** of a functional network is the average of the outreach of all its nodes.

The weighted clustering coefficient ***c_w_(i)*** of a node *i* quantifies the percentage of neighbours of a certain node that, in turn, are neighbours between them, taking into account the weight of the connections:

$$\boldsymbol{c}_{\boldsymbol{w}}\left( \boldsymbol{i} \right)\boldsymbol{=}\frac{\sum_{\boldsymbol{jk}} \boldsymbol{w}_{\boldsymbol{ij}}\boldsymbol{w}_{\boldsymbol{jk}}\boldsymbol{w}_{\boldsymbol{ik}}}{\sum_{\boldsymbol{jk}} \boldsymbol{w}_{\boldsymbol{ij}}\boldsymbol{w}_{\boldsymbol{ik}}}$$

The weighted clustering coefficient ***C_w_*** of a functional network is the average of the weighted clustering coefficient of all its nodes.

The eigenvector centrality ***ev(i)*** of a node *i* measures the importance of a node according to the importance of its neighbours. It is obtained from the eigenvector associated with the largest eigenvalue of the connectivity matrix.

The within-module degree ***z(i)*** of a node *i* measures the importance of a node inside its community. In our case, the community of a node is the lobe the node belongs to:

$$\boldsymbol{z}\left( \boldsymbol{i} \right)\boldsymbol{=}\frac{\boldsymbol{k}_{\boldsymbol{i}}\boldsymbol{(}\boldsymbol{m}_{\boldsymbol{i}}\boldsymbol{)-}\left\langle\boldsymbol{k}_{\boldsymbol{i}}\boldsymbol{(}\boldsymbol{m}_{\boldsymbol{i}}\boldsymbol{)} \right\rangle}{\boldsymbol{\sigma}_{\boldsymbol{k(}\boldsymbol{m}_{\boldsymbol{i}}\boldsymbol{)}}}$$

where $\boldsymbol{k}_{\boldsymbol{i}}\boldsymbol{(}\boldsymbol{m}_{\boldsymbol{i}}\boldsymbol{)}$ is the degree of node *i* inside its community (lobe), and $\left\langle\boldsymbol{k}_{\boldsymbol{i}}\boldsymbol{(}\boldsymbol{m}_{\boldsymbol{i}}\boldsymbol{)} \right\rangle$ and $\boldsymbol{\sigma}_{\boldsymbol{k(}\boldsymbol{m}_{\boldsymbol{i}}\boldsymbol{)}}$ are the average and the standard deviation of the degree inside the community, respectively.

The participation coefficient ***p(i)*** of node *i* quantifies the percentage of links of a node that reach other communities:

$$\boldsymbol{p}\left( \boldsymbol{i} \right)\boldsymbol{=1-}\sum_{\boldsymbol{m}} {\boldsymbol{(}\frac{\boldsymbol{k}_{\boldsymbol{i}}\boldsymbol{(m)}}{\boldsymbol{k}_{\boldsymbol{i}}}\boldsymbol{)}}^{\boldsymbol{2}}$$

where $\boldsymbol{k}_{\boldsymbol{i}}\boldsymbol{(m)}$ is the degree of node *i* inside community *m*.

The average shortest path ***d*** of node *i* is the average of the minimum number of nodes to be visited when going from node *i* to *j*. To obtain *d*, we weight the distances between nodes ***D_ij_***, as the inverse of the elements of the connectivity matrix $\boldsymbol{w}_{\boldsymbol{ij}}$, i.e. ***D_ij_*** *= 1/*$\boldsymbol{w}_{\boldsymbol{ij}}$. Next, we calculate the shortest-path distance between every pair of nodes using the Dijkstra’s algorithm (Dijkstra, 1959). This way, we obtain the shortest-path matrix $\boldsymbol{dis}_{\boldsymbol{ij}}$ (Newman, 2010), and finally the average shortest path ***d*** is obtained as the average of the distance of each node to the rest of the network:

$$\boldsymbol{d=}\frac{\boldsymbol{1}}{\boldsymbol{N(N-1)}}\sum_{i\neq j} {dis}_{ij}$$

The global Efficiency ***Eg*** of a network, first introduced by Latora & Marchiori (2001), overcomes the fact that certain nodes of a network could be isolated from the others, thus leading to infinite distance between them. Mathematically, ***Eg*** is defined as the harmonic mean of the inverse of the shortest paths between all nodes of the network, with ${dis}_{ij}$, being the shortest path between nodes i and j:

$$\boldsymbol{E}_{\boldsymbol{g}}\boldsymbol{=}\frac{\boldsymbol{1}}{\boldsymbol{N(N-1)}}\sum_{i\neq j} \frac{1}{{dis}_{ij}}$$

**S2.- ENTROPY AND COMPLEXITY**

In the matter of dynamical complexity, information plays an important role as a feature that describes the outermost bounds of periodicity, chaos and complexity. In this sense, the Bandt & Pompe (2002) BP method obtains the intrinsic temporal symbol sequences ***{St}*** from the neighbouring steps of a time series (see Fig. S1 for a qualitative explanation). This symbol sequences depend on an embedding dimension *D* = 3**,** 4**,** 5**, …**, which represents the amount of past information, being *D* the number of neighbouring samples. In this way, *D* characterizes each ***{Xt}*** time series along *t* = 1**,** 2**, …,** *M* samples. To do that, ***{Xt}*** is partitioned into (*M* ***-*** *D*) overlapping vectors of dimension *D*.


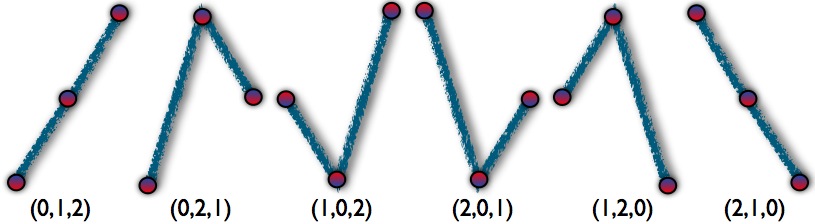


**Figure S1:** Overlapping vectors for the case *D* = 3. *D!* is the number of patterns. This way 3! different types $\pi$ of accessible states are presented. The probability of appearance of the ordinal patterns is contained in *P*. If an ordinal pattern never appears, it is called a forbidden pattern.

The greater the dimension *D*, the more information about the past state of our system, and the longer the vectors are (containing the ordering of a set of *D* samples). Each of the vectors is assigned to a time *t*, sliding the vector at every time step, to get a total of (*M – D + 1*) overlapping vectors. Hence, for each (*M - D*) vector, the position of the lowest value will be assigned the ordinal value zero. The position of the highest value will correspond to (*D - 1*) ordinal value (the highest in the ranking). Thus, the following positions in-between the assigned zero and (*D - 1*) will be assigned by rating the positions of the remaining samples in the respective ordinal values. When all (*M – D + 1*) different order types in ***{St}*** are calculated, it is possible to obtain the probability distribution function (PDF) *P(*$\pi$*)*, quantifying the probability of finding a certain order pattern associated to ***{Xt}:***

$$\boldsymbol{P}\left( \pi\right)\boldsymbol{=}\frac{\boldsymbol{\#}\left\{ \boldsymbol{t} \right|\boldsymbol{t\leq M-D,}\left( \boldsymbol{x}_{\boldsymbol{t+1}}\boldsymbol{, \ldots,}\boldsymbol{x}_{\boldsymbol{t+D}} \right)\boldsymbol{has a type}\pi\boldsymbol{\}}}{\boldsymbol{M-D+1}}$$

In previous equation, $\pi$ is a possible ordinal pattern presented in the sequence ***{St}*** and # is its number of appearances. Note that each ordinal pattern is a permutation of $\pi$ = (0**,**1**,**2**, …,** *D* **-** 1).

In other words, *D!* represents all possible permutations $\pi$ of order *D* of the number of accessible states (M **-** D). As an example, consider the case of *D* = 3 in Fig. 1.8. The number of patterns or accessible states will be *D! = 3! = 6* and the possible patterns $\pi$ will be: **{**(012)**,** (021)**,** (102)**,** (201)**,** (120)**,** (210)**}**. From the former ones, vectors that appear in ***{St}*** are called ordinal patterns of ***{Xt}****,* those that do not appear in ***{St}****,* but belong to the possible accessible states are called forbidden patterns. Finally, the discrete PDF of the ordinal *patterns P =*$p_{j}$***,*** $\forall$ *j = 1****,****2****, …,*** *N* $\wedge$ *N = D!* is calculated. This PDF, obtained from BP method, carries the temporal information of ***{Xt}*** by comparing consecutive samples. In other words, this symbolic technique incorporates the causality effects of a short-memory (of *D* steps) a time series has.

Next, we use the PDF of the ordinal patterns to define the *Normalized Permutation Entropy* *H* (Band & Pompe, 2002):

*Definition S1 — Normalized Permutation Entropy H****[****P****]****. It is given by the ratio between the entropy S****[****P****]*** *of the ordinal patterns and Smax* ***=*** *S****[****Pe****]****, being Pe the uniform probability distribution:*

*H****[****P****] =*** *S****[****P****]/****Smax*

Note that, the normalized permutation entropy *H****[****P****]*** is bounded between [0, 1].

Regarding the finite size effects, the normalized permutation entropy *H* allows to include a uniform distribution *Pe* = **{**1**/**N**,** 1**/**N**, …,** 1**/**N**}** making *H* to be an intensive property. This uniform distribution *Pe*, also maximizes the associate-system information entropy *S[P]*, i.e., *Smax =log(N)= log(D!)*. This way, the amount of disorder *H[P]* based on the information measure *S[P]* associated to *P* is defined as the Permutation Entropy because it runs over all *D!* permutations $\pi$ of order *D*.

On the other hand, the insertion of “a priori" equilibrium distribution **{***Pe***}** as a correction for the associated entropy, leads to a discrimination between two populations. In other words, we need to evaluate the distance between both distributions *P* and *Pe*. This fact makes *S****[****P****]*** not being enough to effectively characterize ***{Xt}*** because there could be some ordinal patterns that belong to *P* as well as to *Pe*. This distance accounts for the “order" of the system when one of the few ordinal patterns emerges as the preferred one. The disequilibrium between statistical populations will be the measure to distinguish this non-Euclidean distance. We quantify the disequilibrium *Q* by adopting some statistical distance *D* between the possible and accessible states of the systems in *P* and the equilibrium distribution *Pe*:

*Definition S2 — Disequilibrium Q****[****P****]****. It evaluates the distance between P and Pe as:*

*Q****[****P****] =*** *Q0 D* ***[****P****,*** *Pe****]***

where *Q0* is a normalization constant leading to 0 $\leq$ *Q****[****P****]*** $\leq$ 1. A Q $\neq$ 0 indicates the existence of preferred states among the accessible ones.

In this way, the disequilibrium *Q[P]*, discriminates ordinal patterns in *P* from the uniform distribution *Pe*. The zero limit or the minimum disequilibrium, implies that the lowest separation of both populations does not distinguish between ordinal patterns coming from both populations. Meanwhile the upper limit, with a high disequilibrium, is related to the fact of the existence of some privileged ordinal patterns in *P*.

Hitherto, both *H* and *Q* give some sense of the understanding of what the dynamical properties of the system are. Nevertheless, we are concerned on evaluating the interplay between the order and disorder of a system. Therefore, it is also desirable to complement these measures with some metric quantifying the complexity of the system.

In this way, Bandt and Pompe define the statistical complexity of a system as:

*Definition S3 — Statistical Complexity* ***C[P]****. This complexity mesures is defined as the product between the permutation entropy* ***H*** *and the disequilibrium* ***Q****:*

*C[P] = H[P] *Q[P]*

With this definition, the statistical complexity ***C*** accomplishes the first requirement since ***H*** and ***Q*** are intensive quantities. The second requirement is also achieved since, by means of ***H*** and ***Q***, we are measuring the disorder of a system and its distance from the equilibrium. Note that the statistical complexity vanishes either if the system is at equilibrium (maximum disorder) or if it is completely ordered (maximal distance from the equilibrium). Figure 1.9 shows a qualitative plot indicating the interplay between the three measures.


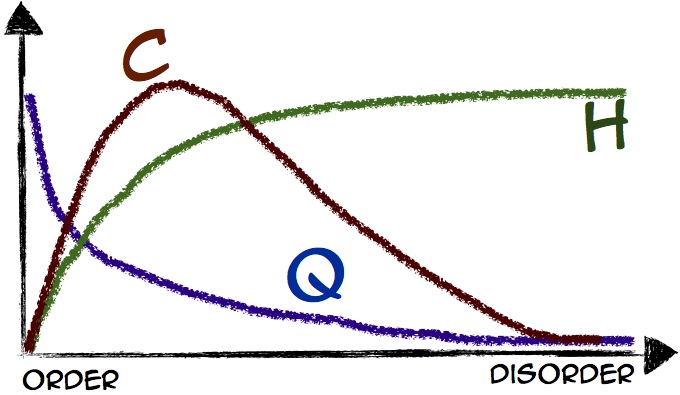


**Figure S2:** Statistical measures based on ordinal patterns. Schematic representation of the interplay between the normalized permutation entropy *H****[****P****]***, disequilibrium *Q****[****P****]*** and the statistical complexity *C****[****P****]***, in terms of a system that range from complete order to complete disorder.

The previous definitions of *H*, *Q* and *C* are usually known as Generalized Statistical Complexity Measures (SCM). SCM capture either, the essential details of the dynamics that allow discerning among different degrees of periodicity and randomness, as well as all possible degrees of stochasticity when the information of ***{Xt}*** is extracted via the BP method. SCM, not only compute randomness, but a wide range of correlation structures, not already offered by a simple entropy analysis.

**S3.- COMPLEMENTARY RESULTS**

**Figure S3.** Differences between high and low CR groups at the node level. Black dots indicate the Euclidean position of the 148 magnetometers (nodes). Circles filled with lilac colour show nodes with significant statistical differences obtained with Mann-Whitney U-test in the: Eigenvector centrality $\Delta\bar{\mathrm{ev}}\left( i \right)$ (**A**), within-module degree z-score $\Delta\bar{z}\left( i \right)$ (**B**) and participation coefficient $\Delta\bar{p}\left( i \right)$ (**C**). Green borders correspond to those nodes that have greater values in the high CR group, and red borders represent those nodes that exhibit greater values in the low CR group. Circle sizes are proportional to the absolute value of the differences between groups. Here we present the results obtained with the permutation test.

| Group | $\bar{S}$ | $\bar{O}$ (**) | $\bar{C}_{w}$ | $\bar{S}_{nn}$ | $\bar{E}_{g}$ | $\bar{d}$ (*) |
| --- | --- | --- | --- | --- | --- | --- |
| *Low* | 13.78 | 13.57 | 0.102 | 13.96 | 0.096 | 12.69 |
| *High* | 12.75 | 12.29 | 0.093 | 12.76 | 0.089 | 14.12 |

**Table S1.** Average network parameters of the low and high CR groups. Specifically, the network strength $\bar{S}$, the outreach $\bar{O}$, the weighted clustering coefficient $\bar{C}_{w}$, the average neighbour strength $\bar{S}_{nn}$, the global efficiency $\bar{E}_{g}$and the average shortest path $\bar{d}$. Asterisks indicate those metrics with statistically significant differences between groups: one asterisk for the parameters passing the rank-sum test and two asterisks for those parameters that also passed the permutation test.

**Figure S4.** Differences of entropy $\bar{H}$(i) between low and high CR groups. In A, we show the mean and standard deviations of the entropy of nodes that have statistical significant differences between groups. ${\bar{H}(i)}^{\mathrm{lowCR}}$ (red squares) is higher than ${\bar{H}(i)}^{\mathrm{highCR}}$(blue circles) for nearly all nodes. In B, we plot the position of nodes with statistical differences. Node sizes are proportional to $\left| \Delta\bar{H}(i)={\bar{H}(i)}^{\mathrm{highCR}}{-\bar{H}(i)}^{\mathrm{lowCR}} \right|$. Red borders represent$\Delta\bar{H}(i)<0$, otherwise node borders are green. In this case, significant statistical differences were obtained with Mann-Whitney U-test.

**Figure S5.** Differences of complexity $\bar{C}$(i) between the Low and High CR groups. In A, we show the mean and standard deviations of the entropy of nodes that have statistical significant differences between groups. ${\bar{C}(i)}^{\mathrm{lowCR}}$ (red squares) is lower than ${\bar{C}(i)}^{\mathrm{highCR}}$(blue circles) for nearly all nodes. In B, we plot the position of nodes with statistical differences. Node sizes are proportional to $\left| \Delta\bar{C}(i)={\bar{C}(i)}^{\mathrm{highCR}}{-\bar{C}(i)}^{\mathrm{lowCR}} \right|$. Green borders represent$\Delta\bar{C}\left( i \right)>0$, otherwise node borders are red. Statistical significant differences were obtained with Mann-Whitney U-test.

**Figure S6.** Complexity-Entropy Diagram. Diagram of subjects’ average complexity vs. entropy for all 148 nodes (i.e., each point represents a node). Red squares correspond to low CR group and blue circles to the high CR group. The inset shows only nodes with statistical significant differences using the permutation test. Specifically, these nodes are the channels that have statistical differences when comparing both the mean entropies and complexities of high vs. low CR groups at node level. Statistical significant differences were obtained with Mann-Whitney U-test.

| $r^{2}$ | $\left( \bar{S},\bar{H} \right)^{Low}$ | ${(\bar{S},\bar{H})}^{High}$ | ${(\bar{S},\bar{C})}^{Low}$ | ${(\bar{S},\bar{C})}^{High}$ | ${(\bar{C}}_{w},\bar{H})^{Low}$ | ${(\bar{C}}_{w},\bar{H})^{High}$ | ${(\bar{C}}_{w},\bar{C})^{Low}$ | ${(\bar{C}}_{w},\bar{C})^{High}$ |
| --- | --- | --- | --- | --- | --- | --- | --- | --- |
| *Lin.* | 0.5833 | 0.4105 | 0.6486 | 0.4902 | 0.5719 | 0.3423 | 0.6417 | 0.4246 |
| *Pol.* | 0.5833 | 0.4075 | 0.7124 | 0.5159 | 0.5713 | 0.3332 | 0.7055 | 0.4733 |

**Table S2.** Coefficient $r^{2}$ for the correlations of Fig. 8 of the main text. First (second) row represent the linear (second order polynomic) fit. Columns represent the $r^{2}$ of both averaged variables (structural and dynamical) for the low and high CR groups. Columns 1 and 2 are for Fig. 8A, columns 3 and 4 are for Fig. 8B, columns 5 and 6 are for Fig. 8C. The last two columns are for Fig. 8D. Permutation test and rank-sum test rejected Ho with p<0.001.

**REFERENCES**

Bandt, C. & Pompe, B. Permutation Entropy: A Natural Complexity Measure for Time Series. *Phys. Rev. Lett.* **88**, 174102 (2002).

Dijkstra, E.W. A note on two problems in connexion with graphs. *Numerische Mathematik* **1**, 269–271 (1959).

Latora, V. & Marchiori, M. Efficient behavior of small-world networks*. Physical Review Letters* **87**, 198701 (2001).

Newman, M. E. J. Networks: An introduction. (Oxford University Press, 2010).

Garibotto,V., Borroni,B., Sorbi,S., Cappa,S.F., Padovani,A., and Perani,D.(2011). Education and occupation provide reserve in both ApoE ε4 carrier and non carrier patients with probable Alzheimer’s disease. Neurol.Sci. 33, 1037–1042. doi:10.1007/s10072-011-0889-5
